# Lipopolysaccharide induces HIF-1α accumulation *via* MAPK p38–mediated mRNA stabilization and dexamethasone-sensitive protein stabilization

**DOI:** 10.1016/j.jbc.2025.111094

**Published:** 2025-12-23

**Authors:** Chloe Lockwood, Kalbinder K. Daley, John D. O’Neil, Katherine J. Heighes, Sally A. Clayton, Andrew R. Clark

**Affiliations:** 1 Department of Inflammation and Ageing, School of Infection, Inflammation and Immunology, College of Medicine and Health, University of Birmingham, Edgbaston, United Kingdom; 2 Department of Immunology and Immunotherapy, School of Infection, Inflammation and Immunology, College of Medicine and Health, University of Birmingham, Edgbaston, United Kingdom

**Keywords:** glucocorticoid, macrophage, post-transcriptional regulation, tristetraprolin, MAPK p38, aerobic glycolysis

## Abstract

In macrophages, the hypoxia-inducible transcription factor 1α (HIF-1α) can be activated under normoxic conditions by proinflammatory agonists such as lipopolysaccharide (LPS). This noncanonical HIF-1α activation allows macrophages to accommodate rapidly changing demands for energy and biosynthetic precursors in the face of an immune challenge. Alterations in HIF-1α hydroxylation and proteolysis have been implicated in the response, but the involvement of other signaling mechanisms and pathways is unclear. Here, we use genetic and pharmacological approaches to show that LPS-induced HIF-1α accumulation in primary macrophages is dependent on mitogen-activated protein kinase p38 and mediated by the phosphorylation and inactivation of tristetraprolin, an mRNA destabilizing protein that targets *Hif1a* mRNA for degradation. We previously reported that the glucocorticoid dexamethasone inhibits LPS-induced HIF-1α accumulation and metabolic reprogramming in primary macrophages. Here, we tested and disproved the hypothesis that dexamethasone exerts this effect *via* the mitogen-activated protein kinase p38 inactivator dual specificity phosphatase 1. Hence, two novel mechanisms critically regulate HIF-1α activation in LPS-treated macrophages: a p38-dependent mechanism that operates at the post-transcriptional level to control *Hif1a* mRNA stability, and a glucocorticoid-sensitive mechanism that operates at the post-translational level to control HIF-1α protein stability. Combined targeting of these two mechanisms may exert therapeutic effects in contexts where HIF-1α contributes to immune-mediated inflammatory pathology.

The hypoxia-inducible transcription factor 1α (HIF-1α) plays a central role in cellular adaptation to oxygen insufficiency, coordinating profound metabolic changes to ensure continued generation of ATP when mitochondrial function is impaired (1, 2). The mechanisms by which decreasing oxygen availability leads to the activation of HIF-1α are well understood. When oxygen is freely available, HIF-1α protein is constitutively produced but then hydroxylated by oxygen-dependent prolyl hydroxylase (PHD) enzymes. Hydroxylated HIF-1α is then recognized and ubiquitinated by the von Hippel–Lindau (VHL) ubiquitin ligase complex, and the lysine 48-linked polyubiquitin chains mark HIF-1α for rapid degradation by the proteasome. When cellular oxygen levels decrease, HIF-1α protein accumulates because of impairment of hydroxylation and degradation processes. HIF-1α then dimerizes with its constitutively expressed binding partner HIF-1β, binds to hypoxia-responsive elements that usually contain the consensus sequence (A/G)CGTG and activates transcription. The genes that are regulated in this manner include several mediators of glycolytic metabolism, such as the glucose transporter *Slc2a1* (GLUT1) and the lactate transporter *Slc16a3* (MCT4). The HIF-1α-mediated shift to glycolytic metabolism enables cells to generate ATP independently of oxidative phosphorylation in mitochondria.

HIF-1α can also be activated by the proinflammatory bacterial cell wall component lipopolysaccharide (LPS) in myeloid cells, independently of oxygen availability (3, 4, 5). LPS-induced HIF-1α activation promotes a shift to glycolytic metabolism, regardless of oxygen sufficiency (the Warburg effect), permitting cells to rapidly increase ATP generation. HIF-1α also promotes proinflammatory gene expression, migration, and antimicrobial functions, which are essential for protection against pathogens (5, 6, 7, 8, 9). However, the mechanism(s) by which LPS and other proinflammatory factors promote HIF-1α accumulation are not fully understood. Some studies demonstrated that NF-κB activated transcription of the *Hif1a* gene (10, 11). Others have shown that disruption of the mitochondrial tricarboxylic acid cycle leads to accumulation of metabolites that impair PHD function, promoting HIF-1α accumulation at the post-translational level (12, 13). Mitogen-activated protein kinase (MAPK) p38 was implicated in the LPS-induced accumulation of HIF-1α protein in mouse neutrophils and human monocyte–derived dendritic cells (14, 15). Disruption of the *Dusp1* (dual specificity phosphatase 1) gene enhanced and prolonged the activation of MAPK p38 by LPS in murine macrophages (16, 17, 18, 19) and augmented LPS-induced glycolytic metabolism (20, 21). One group of investigators found increased expression of HIF-1α protein in LPS-treated *Dusp1*^*−/−*^ mouse macrophages (19). Another group found no change of HIF-1α expression in LPS-treated *Dusp1*^*−/−*^ mouse macrophage and instead implicated MAPK p38–mediated phosphorylation of the glycolysis regulator PFKFB3 (21). MAPK p38 also contributes to the accumulation of HIF-1α in response to hypoxia, ischemia–reperfusion, and other oxidative stresses in mouse embryonic fibroblasts (22), keratinocytes (23) and various tumor cell lines (6, 24, 25). To our knowledge, only one article has directly addressed the mechanism by which MAPK p38 regulates HIF-1α expression. Kwon *et al*. (24) proposed that MAPK p38–dependent phosphorylation impairs the interaction of hydroxylated HIF-1α with the VHL complex in ischemic pancreatic cancer cells, preventing its degradation by the proteasome. This mechanism has not yet been corroborated in other cell types.

We recently reported that the glucocorticoid dexamethasone destabilized HIF-1α protein in primary macrophages, preventing the LPS-induced glycolytic shift (26). Dexamethasone exerts many of its anti-inflammatory effects by increasing the expression of DUSP1 and thereby downregulating the MAPK p38 pathway (16, 27, 28, 29, 30, 31). DUSP1 exerts anti-inflammatory effects by modulating the phosphorylation status and activity of the mRNA-destabilizing factor tristetraprolin (TTP) (32, 33). MAPK p38–dependent phosphorylation both stabilizes and inactivates TTP. In *Dusp1*^*−/−*^ mouse macrophages, where LPS-induced p38 activation is dysregulated, TTP accumulates in its phosphorylated and inactive forms, accompanied by increased expression of genes that are negatively regulated by TTP, for example, *Tnf* and *Ptgs2* (32, 34). Several groups have independently reported that TTP destabilizes *Hif1a* mRNA and reduces expression of HIF-1α protein (35, 36, 37).

Drawing together these observations, we proposed two distinct hypotheses to explain how dexamethasone inhibits HIF-1α accumulation and glycolytic metabolism in LPS-activated macrophages. Dexamethasone increases the expression of DUSP1 and thereby inhibits MAPK p38 signaling. According to the first hypothesis, this reduction of MAPK p38 activity favors the activation of TTP and destabilization of *Hif1a* mRNA (*i*.*e*., post-transcriptional regulation). According to the second hypothesis, lower p38 activity favors VHL-mediated ubiquitination and proteasome-mediated degradation of HIF-1α protein (*i*.*e*., post-translational regulation). To test these hypotheses, we used various pharmacological and genetic approaches in both primary mouse bone marrow–derived macrophages (BMDMs) and primary human monocyte-derived macrophages (MDMs).

## Results

**Figure 1:**
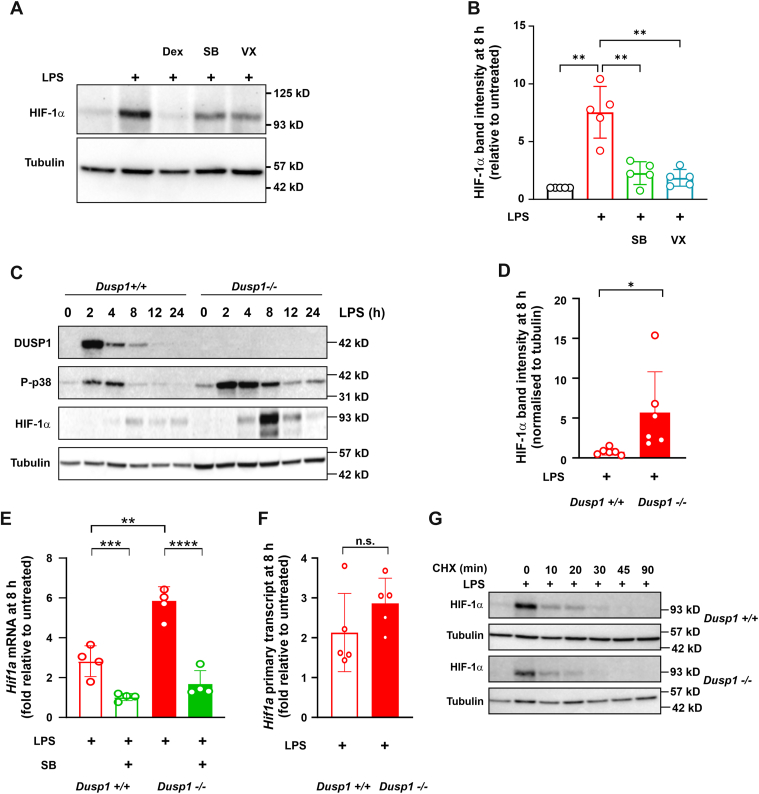
**MAPK p38 regulates HIF-α accumulation in LPS-activated mouse BMDMs**. Here and throughout, treatment conditions are indicated by different colors, and genotypes (where appropriate) are indicated by different shadings. *A*, mouse BMDMs were treated for 8 h with 10 ng/ml LPS alone or in combination with 100 nM dexamethasone (Dex), 1 μM SB202190 (SB), or 1 μM VX-745 (VX). HIF-1α and α-tubulin were detected by Western blotting. *B*, quantification of HIF-1α expression (mean ± SD, normalized to α-tubulin and expressed relative to untreated control; five independent experiments as in *A*; one-way ANOVA with Dunnett’s correction for multiple comparison). *C*, *Dusp1*^+/+^ and *Dusp1*^−/−^ BMDMs were treated with 10 ng/ml LPS for the indicated times, and DUSP1, phospho-p38, HIF-1α, and α-tubulin were detected by Western blotting. Representative blots from one of three independent experiments. *D*, quantification of HIF-1α expression in *Dusp1*^+/+^ and *Dusp1*^−/−^ BMDMs 8 h after addition of LPS (mean ± SD, normalized to α-tubulin; six independent experiments; unpaired *t* test). *E*, *Dusp1*^+/+^ and *Dusp1*^−/−^ BMDMs were treated with LPS for 8 h in the absence or the presence of 1 μM SB. *Hif1a* mRNA was measured by quantitative PCR (normalized to unstimulated control; mean ± SD of four independent experiments; two-way ANOVA). *F*, *Dusp1*^+/+^ and *Dusp1*^−/−^ BMDMs were treated with LPS for 8 h, and *Hif1a* primary transcript was measured by quantitative PCR (normalized to unstimulated control; mean ± SD of five independent experiments; unpaired two-tailed *t* test). No PCR product was detected if the reverse transcriptase step was omitted, indicating the absence of contaminating genomic DNA. *G*, *Dusp1*^+/+^ and *Dusp1*^−/−^ BMDMs were treated with LPS for 8 h, and then cycloheximide (CHX; 5 μg/ml) was added at *t* = 0, and protein was harvested after the indicated times. HIF-1α and α-tubulin were detected by Western blotting. Loading was adjusted for approximately equal HIF-1α band intensity. Representative blot of one of three independent experiments. ns, *p* > 0.05; ∗*p* < 0.05; ∗∗*p* < 0.01; ∗∗∗*p* < 0.005; and ∗∗∗∗*p* < 0.001. BMDM, bone marrow–derived macrophage; DUSP1, dual specificity phosphatase 1; HIF-α, hypoxia-inducible transcription factor 1α; LPS, lipopolysaccharide; MAPK, mitogen-activated protein kinase; ns, not significant.

MAPK p38 has previously been implicated in the accumulation of HIF-1α protein in response to various stimuli. To test the involvement of MAPK p38 in LPS-induced HIF-1α expression, primary mouse BMDMs were pretreated with two chemically distinct p38 inhibitors (SB202190 and VX-745) for 30 min prior to the addition of LPS. Dexamethasone was used here as a positive control, which we have previously shown to inhibit LPS-induced HIF-1α accumulation (26). The LPS-induced accumulation of HIF-1α protein was also significantly impaired by both p38 inhibitors (Fig. 1, *A* and *B*). For each inhibitor, the dose dependencies of MAPK p38 inhibition and HIF-1α protein downregulation were similar (Fig. S1). Although the two MAPK p38 inhibitors differed in their dose responses, their effects on MAPK p38 activity and HIF-1α expression were comparable at 1 μM concentration. To confirm these findings, we investigated *Dusp1*^*−/−*^ BMDMs, in which the absence of an important negative feedback factor results in dysregulation of MAPK p38 signaling (16). Enhanced and prolonged LPS-induced activation of MAPK p38 in *Dusp1*^*−/−*^ BMDMs was confirmed (Fig. 1*C*). This dysregulated signaling was accompanied by increased expression of HIF-1α protein, which was particularly evident at the 8 h time point (Fig. 1, *C* and *D*). Expression of *Hif1a* mRNA was dependent on MAPK p38 and enhanced in *Dusp1*^*−/−*^ BMDMs (Fig. 1*E*). However, *Dusp1*^*+/+*^ and *Dusp1*^*−/−*^ BMDMs did not differ in *Hif1a* transcription (estimated by measurement of primary transcript) (Fig. 1*F*). The stability of HIF-1α protein also did not differ between *Dusp1*^*+/+*^ and *Dusp1*^*−/−*^ BMDMs, as demonstrated by cycloheximide chases (Fig. 1*G*). These observations suggest that the DUSP1-p38 pathway may regulate HIF-1α accumulation at the level of *Hif1a* mRNA stability rather than transcription or HIF-1α protein stability.

**Figure 2:**
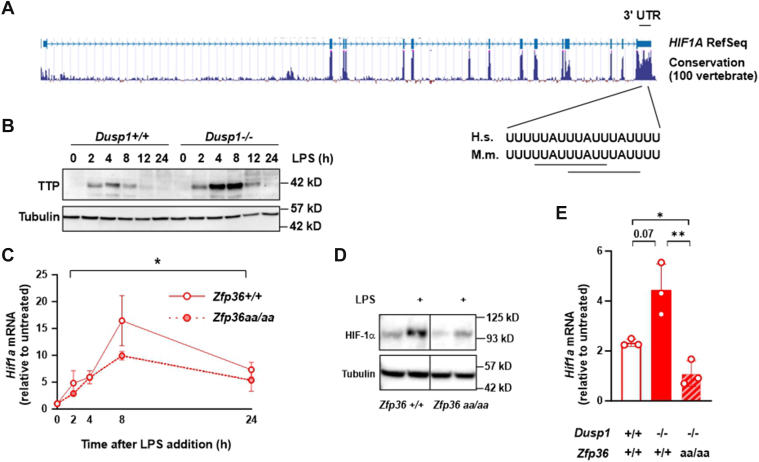
**DUSP1 controls HIF-1α expression by modulating the phosphorylation of TTP**. *A*, evolutionary conservation of the *HIF1A* 3′ UTR between 100 vertebrate species (from UCSC Genome Browser). Conservation of consensus TTP binding sites in mouse *Hif1a* and human *HIF1A* 3′UTRs. *B*, *Dusp1*^*+/+*^ and *Dusp1*^*−/−*^ BMDMs were treated with 10 ng/ml LPS for the indicated times. TTP and α-tubulin were detected by Western blotting. Representative of two independent experiments. *C*, *Zfp36*^*+/+*^ and *Zfp36*^*aa/aa*^ BMDMs were treated with 10 ng/ml LPS for the indicated times, and *Hif1a* mRNA abundance was measured by quantitative PCR (mean ± SD fold change relative to untreated control; three independent experiments; two-way ANOVA with Dunnett’s correction; *asterisk* indicates significant effect of genotype). *D*, *Zfp36*^*+/+*^ and *Zfp36*^*aa/aa*^ BMDMs were left untreated or stimulated with 10 ng/ml LPS for 8 h. HIF-1α and α-tubulin were detected by Western blotting. Representative of three independent experiments. *Vertical line* represents splicing of different sections of a single gel image. *E*, wildtype (*Dusp1*^*+/+*^:*Zfp36*^*+/+*^), DUSP1 knockout (*Dusp1*^*−/−*^:*Zfp36*^*+/+*^), and double-modified (*Dusp1*^*−/−*^:*Zfp36*^*aa/aa*^) BMDMs were stimulated with 10 ng/ml LPS for 4 h. Then *Hif1a* mRNA abundance was measured by quantitative PCR (mean ± SD fold change relative to untreated control; three independent experiments; one-way ANOVA with Dunnett’s correction). ∗*p* < 0.05; ∗∗*p* < 0.01. BMDM, bone marrow–derived macrophage; DUSP1, dual specificity phosphatase 1; HIF-1α, hypoxia-inducible transcription factor 1α; LPS, lipopolysaccharide; TTP, tristetraprolin; UCSC, University of California, Santa Cruz.

*Hif1a* mRNA was reported to be targeted for degradation by the RNA-destabilizing protein TTP (35, 36, 37). As previously noted (36), the *Hif1a* 3′ UTR sequence is strongly conserved between vertebrate species and contains an adenosine-/uridine-rich element with two overlapping consensus TTP-binding sites (UUAUUUAUU, highlighted by *horizontal lines* in Figure 2*A*). This arrangement of overlapping motifs is typical of high-affinity TTP-binding sites and identical to sites that are recognized by TTP in *Tnf* and *Ptgs2* (COX-2) mRNAs (38). MAPK p38 activates its downstream effector kinase MK2, which then phosphorylates TTP at two critical serine residues—Ser52 and Ser178 in mouse. These phosphorylations both stabilize and inactivate the TTP protein. Therefore, in *Dusp1*^*−/−*^ BMDMs, TTP protein accumulates in an inactivate form that is accompanied by increased expression of target mRNAs (32). The accumulation of TTP protein in *Dusp1*^*−/−*^ BMDMs was confirmed (Fig. 2*B*). This provides a hypothetical mechanism by which the MAPK p38 pathway could act at the post-transcriptional level to promote *Hif1a* mRNA accumulation; namely, p38-dependent phosphorylation and inactivation of TTP, resulting in stabilization of *Hif1a* mRNA. To test this hypothesis, we used a knock-in mouse strain in which Ser52 and Ser178 codons of the endogenous mouse TTP locus (formally *Zfp36*) were mutated to alanine codons. In BMDMs derived from this strain (*Zfp36*^*aa/aa*^), endogenous TTP protein cannot be phosphorylated and inactivated downstream of the MAPK p38 pathway (39). It therefore behaves as a constitutive negative regulator of target mRNAs, including large numbers of inflammatory mediators (39, 40, 41, 42). The LPS-induced expression of *Hif1a* mRNA was impaired in *Zfp36*^*aa/aa*^ BMDMs; this effect was again most evident at the 8 h time point (Fig. 2*C*). Expression of HIF-1α protein was also reduced by approximately 60% in *Zfp36*^*aa/aa*^ BMDMs (Fig. 2*D*; also see quantification in Fig. 4*A*). To test whether the overexpression of *Hif1a* mRNA in *Dusp1*^*−/−*^ BMDMs was dependent on the phosphorylation of TTP, we made use of a double genetically modified strain in which the disruption of the *Dusp1* locus was combined with the substitution of Ser52 and Ser178 codons of the *Zfp36* locus. In macrophages derived from this *Dusp1*^*−/−*^:*Zfp36*^*aa/aa*^ strain, the expression of TTP-regulated inflammatory mediators remains low. Although MAPK p38 signaling is elevated, this does not lead to increased gene expression because the critical sites of p38-dependent phosphorylation of TTP are absent (32). *Dusp1* gene disruption caused an increase in LPS-induced expression of *Hif1a* mRNA (Fig. 2*E*). Although here the increase did not achieve statistical significance (n = 3; *p* = 0.07), the previous experiment (Fig. 1*E*) clearly evidenced the increased expression of *Hif1a* mRNA in *Dusp1*^*−/−*^ BMDMs. Expression of *Hif1a* mRNA was significantly lower in *Dusp1*^*−/−*^:*Zfp36*^*aa/aa*^ BMDMs than in *Dusp1*^*−/−*^ BMDMs, suggesting that DUSP1 regulates the expression of HIF-1α *via* the phosphorylation of TTP. We have previously shown that DUSP1 similarly regulates expression of proinflammatory genes, such as *Tnf* and *Ptgs2*, by controlling TTP phosphorylation (32).

**Figure 3:**
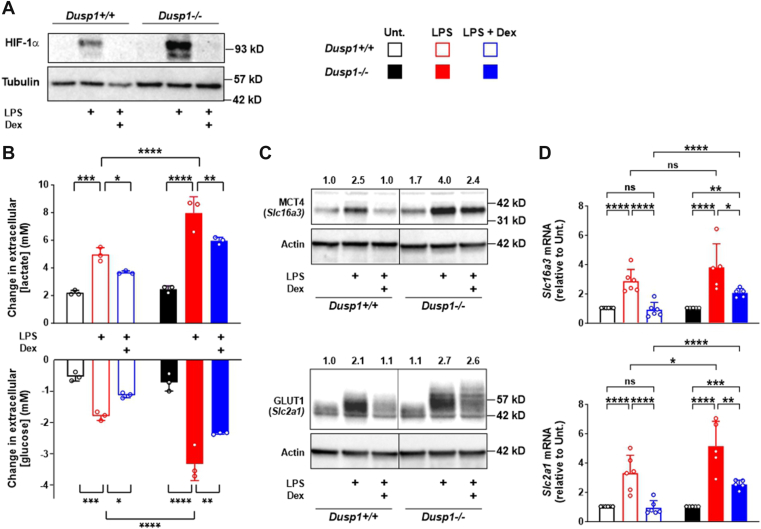
**Dexamethasone (Dex)-mediated inhibition of LPS-induced glycolysis is not dependent on DUSP1**. *A*, *Dusp1*^+/+^ and *Dusp1*^−/−^ BMDMs were treated for 8 h with 10 ng/ml LPS alone or in combination with 100 nM Dex. HIF-1α and α-tubulin were detected by Western blotting. Representative of six independent experiments. *B*, *Dusp1*^+/+^ and *Dusp1*^−/−^ BMDMs were treated for 24 h with 10 ng/ml LPS alone or in combination with 100 nM Dex. Change in extracellular concentration of lactate (*upper*) or glucose (*lower*) (mean ± SD; three independent experiments; two-way ANOVA with Sidak’s correction). *C*, *Dusp1*^*+/+*^ and *Dusp1*^*−/−*^ BMDMs were treated as in (*B*), and whole-cell lysates were blotted for MCT4 (*upper*) or GLUT1 (*lower*), with actin as a loading control. Numbers above the blots are mean protein quantities from three independent experiments, normalized against actin and untreated *Dusp1*^*+/+*^ control. *Vertical line* represents splicing of different sections of a single gel image. *D*, expression of *Slc16a3* and *Slc2a1* mRNA was measured by RT–quantitative PCR after 24 h of treatment as indicated (mean fold change relative to unstimulated control ± SD; five to six independent experiments; two-way ANOVA with Tukey’s correction). ns, *p* > 0.05; ∗*p* < 0.05; ∗∗*p* < 0.01; ∗∗∗*p* < 0.005; and ∗∗∗∗*p* < 0.001. BMDM, bone marrow–derived macrophage; DUSP1, dual specificity phosphatase 1; GLUT1, glucose transporter 1; HIF-1α, hypoxia-inducible transcription factor 1α; LPS, lipopolysaccharide; MCT4, monocarboxylate transporter 4.

**Figure 4:**
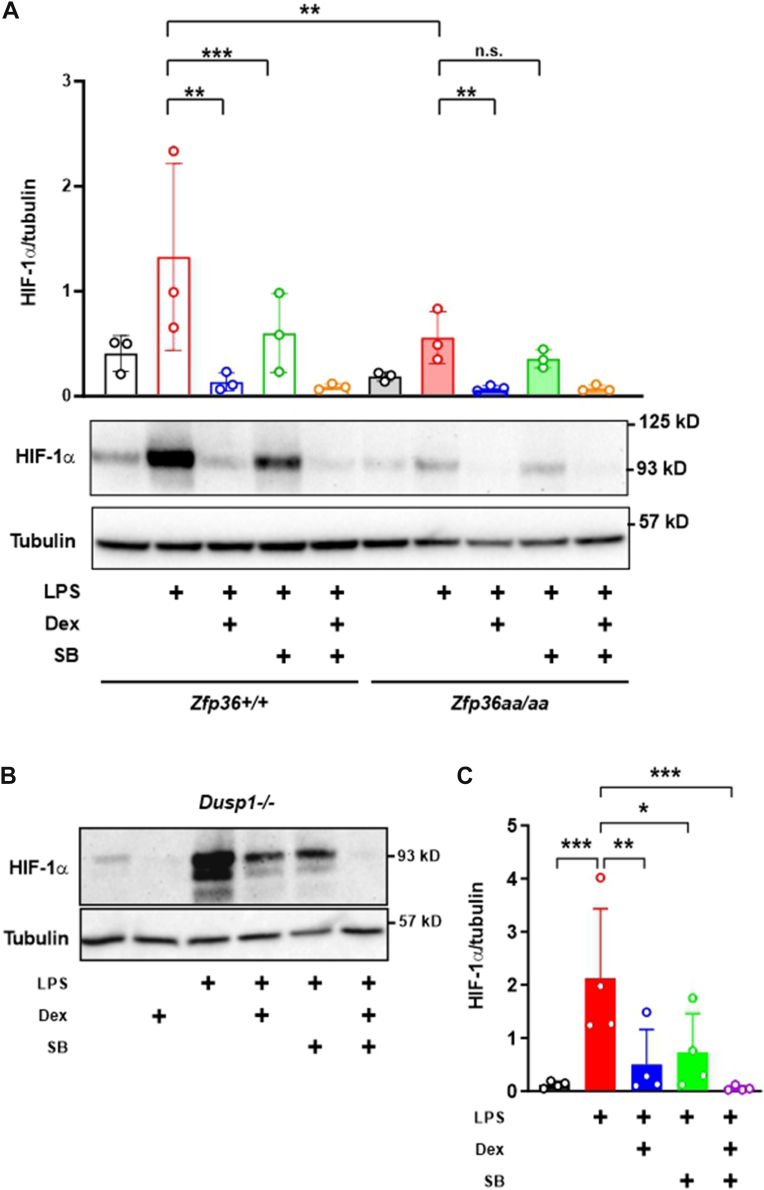
**Dexamethasone impairs HIF-1α expression independently of MAPK p38 and TTP phosphorylation**. *A*, *Zfp36*^*+/+*^ and *Zfp36*^*aa/aa*^ BMDMs were treated for 8 h with combinations of 10 ng/ml LPS, 100 nM dexamethasone (Dex), and 1 μM SB202190 (SB) as shown. HIF-1α and α-tubulin were detected by Western blotting. The graph shows the mean ± SD of HIF-1α/α-tubulin ratio from three independent experiments (two-way ANOVA with Tukey’s correction). *B*, *Dusp1*^*−/−*^ BMDMs were treated for 8 h with combinations of 10 ng/ml LPS, 100 nM Dex, and 1 μM SB as shown. HIF-1α and α-tubulin were detected by Western blotting. *C*, mean ± SD HIF-1α protein expression (normalized against α-tubulin) in four independent experiments using *Dusp1*^*−/−*^ BMDMs treated as in (*B; one-way ANOVA with Dunnett's corection*). ns *p* > 0.05; ∗*p* < 0.05; ∗∗*p* < 0.01; and ∗∗∗*p* < 0.005. BMDM, bone marrow–derived macrophage; HIF-1α, hypoxia-inducible transcription factor 1α; LPS, lipopolysaccharide; MAPK, mitogen-activated protein kinase; TTP, tristetraprolin.

Dexamethasone can exert anti-inflammatory effects by inducing *Dusp1* gene expression and thereby negatively regulating the MAPK p38 pathway (16, 27, 43). We hypothesized that DUSP1 similarly contributes to the dexamethasone-mediated inhibition of HIF-1α expression and downstream metabolic reprogramming. To test this hypothesis, we analyzed the effects of dexamethasone on LPS-induced HIF-1α accumulation, glycolysis, and expression of HIF-1α-regulated genes of the glycolytic pathway in wildtype and *Dusp1*^*−/−*^ BMDMs. LPS-induced HIF-1α protein accumulation was enhanced in *Dusp1*^*−/−*^ BMDMs but remained sensitive to dexamethasone (Fig. 3*A*). LPS-induced lactate secretion and glucose consumption, hallmarks of glycolytic metabolism, were increased in *Dusp1*^*−/−*^ BMDMs but remained sensitive to dexamethasone (Fig. 3*B*). Expression of glucose and lactate transporters is rate limiting for glycolysis (44). In murine macrophages, the glucose transporter GLUT1 and the monocarboxylate transporter MCT4 are critical mediators of glucose uptake and lactate secretion and are encoded by HIF-1α-dependent genes (*Slc2a1* and *Slc16a3*, respectively) (26, 45, 46). LPS-induced expression of MCT4 protein was enhanced in *Dusp1*^*−/−*^ BMDMs (Fig. 3*C*), although the increase in expression of *Slc16a3* mRNA at the 24 h time point did not reach statistical significance (Fig. 3*D*). LPS-induced expression of GLUT1 protein was also enhanced in *Dusp1*^*−/−*^ BMDMs (Fig. 3*C*), accompanied by a significant increase in expression of *Slc2a1* mRNA (Fig. 3*D*). Importantly, dexamethasone retained its ability to impair the expression of GLUT1 and MCT4 protein, *Slc2a1* and *Slc16a3* mRNA in *Dusp1*^*−/−*^ BMDMs. Expression of a third, HIF-1α-dependent glycolytic mRNA, *Tpi1*, displayed a similar pattern of expression in *Dusp1*^*−/−*^ BMDMs (Fig. S2).

Consistent with the lower expression of *Hif1a* mRNA in *Zfp36*^*aa/aa*^ BMDMs, LPS-induced HIF-1α protein expression was also reduced in *Zfp36*^*aa/aa*^ BMDMs but remained sensitive to dexamethasone (Fig. 4*A*). Interestingly, HIF-1α expression was not significantly affected by the p38 inhibitor SB202190 in *Zfp36*^*aa/aa*^ BMDMs. Together, these results show that the MAPK p38 pathway regulates HIF-1α expression and upregulation of glycolytic metabolism *via* the phosphorylation of TTP. Although dexamethasone can induce the expression of DUSP1 and thereby inhibit MAPK p38 in BMDMs (16), the effects of dexamethasone on LPS-induced HIF-1α activation and HIF-1α-dependent gene expression are not strictly dependent on the expression of DUSP1 or the dephosphorylation of TTP.

If dexamethasone and SB202190 reduce the expression of HIF-1α by distinct mechanisms, one might expect the two treatments to exert additive effects. This was difficult to assess in wildtype BMDMs because the powerful inhibitory effect of dexamethasone reduced HIF-1α levels to near undetectable. We therefore used *Dusp1*^*−/−*^ BMDMs, in which the higher expression of HIF-1α protein allowed us to look for additive effects of the two reagents. The LPS-induced expression of HIF-1α was reduced by either dexamethasone or SB202190 in isolation and eliminated by the two reagents in combination (Fig. 4*B*). Again, this supports the argument that dexamethasone impairs HIF-1α expression by a mechanism other than modulation of p38 and TTP.

**Figure 5:**
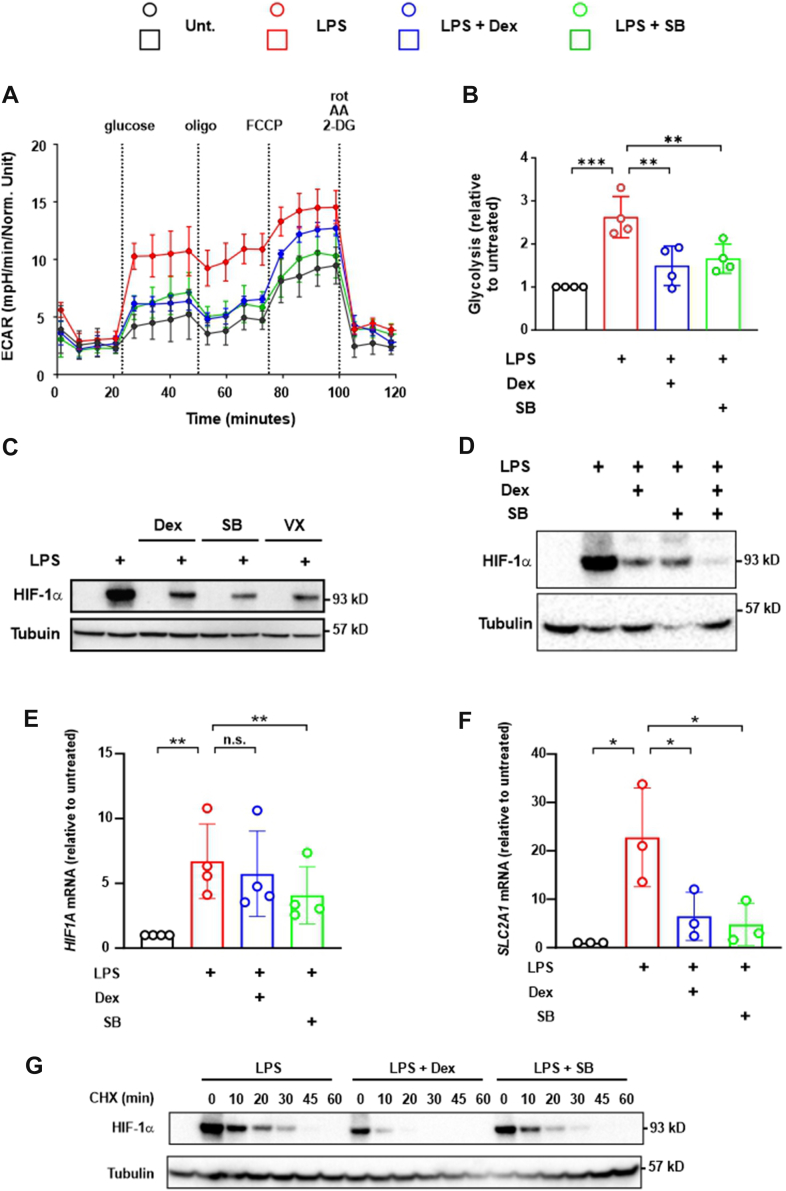
**In human MDMs, MAPK p38 regulates the LPS-induced glycolytic response at the level of *HIF1A* mRNA expression**. *A*, MDMs were treated for 24 h with combinations of 10 ng/ml LPS, 100 nM dexamethasone (Dex), and 1 μM SB202190 (SB) as shown, then glycolysis was measured by Seahorse XFe96 Mito + Glyco stress test (see the *Experimental procedures* section). *B*, mean ± SD fold change in glycolysis relative to unstimulated control from four independent experiments as in *A*; two-way ANOVA with Dunnett’s correction. *C* and *D*, MDMs were treated for 8 h with combinations of 10 ng/ml LPS, 100 nM Dex, 1 μM SB, and 1 μM VX-475 (VX) as shown. HIF-1α and α-tubulin were detected by Western blotting. Representative of three (*C*) or four (*D*) independent experiments. *E* and *F*, MDMs were treated with 10 ng/ml LPS ± 100 nM Dex or 1 μM SB for 8 h (peak of *HIF1A* expression) or 12 h (peak of *SLC2A1* expression). Fold change ± SD of *HIF1A* (*E*) or *SLC2A1* (*F*) mRNA abundance relative to unstimulated control; three (*F*) or four (*E*) independent experiments; one-way ANOVA with Dunnett’s correction. *G*, MDMs were stimulated for 8 h with 10 ng/ml LPS in the absence or the presence of 100 nM Dex or 1 μM SB. Cycloheximide (CHX) was added at *t* = 0, and cells were harvested at the time points shown. HIF-1α and α-tubulin were detected by Western blotting. Representative of three independent experiments. ns, *p* > 0.05; ∗*p* < 0.05; ∗∗*p* < 0.01; ∗∗∗*p* < 0.005; and ∗∗∗∗*p* < 0.001. HIF-1α, hypoxia-inducible transcription factor 1α; LPS, lipopolysaccharide; MAPK, mitogen-activated protein kinase; MDM, monocyte-derived macrophage; ns, not significant.

We then tested the effects of MAPK p38 inhibitors in human MDMs. SB202190 and dexamethasone inhibited LPS-induced glycolysis to similar extents (Fig. 5, *A* and *B*) (26). As in mouse BMDMs, both SB202190 and the structurally unrelated MAPK p38 inhibitor VX-745 reduced the LPS-induced expression of HIF-1α protein in human MDMs (Fig. 5*C*). As in mouse BMDMs, dexamethasone and SB202190 cooperated with one another to reduce the expression of HIF-1α protein (Fig. 5*D*). SB202190 impaired LPS-induced expression of *HIF1A* mRNA, whereas dexamethasone did not do so (Fig. 5*E*). Both reagents impaired the LPS-induced expression of the HIF-1α target gene *SLC2A1* (Fig. 5*F*). As previously reported (26) dexamethasone destabilized HIF-1α protein, reducing its half-life by almost fourfold from 8.54 min (95% confidence interval: 7.42–9.85 min) to 2.41 min (95% confidence interval: 1.83–2.89 min) (Fig. 5*G*). Under the same conditions, SB202190 did not affect HIF-1α protein stability (Fig. 5*G*). Overall, the results presented show that dexamethasone and SB202190 regulate HIF-1α protein accumulation at distinct levels: dexamethasone at the post-translational level and SB202190 at the level of mRNA stability.

## Discussion

The MAPK p38 signaling pathway positively regulates glycolysis in myeloid cells (14, 15, 20, 21). As a master regulator of glycolysis, HIF-1α is a prime candidate mediator of this effect. Indeed, several articles have described regulation of HIF-1α function or expression by the MAPK p38 signaling pathway in different contexts (6, 14, 15, 19, 22, 23, 24, 25, 36, 47, 48, 49), but few have addressed sites or mechanisms of action. A selective MAPK p38 inhibitor impaired the hypoxia-induced activation of a hypoxia-responsive element reporter or a reporter controlled by a Gal4-HIF-1α transcriptional activation domain (TAD) fusion protein in a liver cancer cell line (49). The same inhibitor did not affect HIF-1α protein expression, suggesting that MAPK p38 directly or indirectly regulates the function of the HIF-1α TAD. We cannot formally rule out that MAPK p38 also regulates HIF-1α TAD function in macrophages. However, MAPK p38–mediated regulation of HIF-1α expression levels in these cells may be sufficient to explain downstream changes in gene expression and metabolism. Others reported that MAPK p38–mediated phosphorylation of HIF-1α impaired its interaction with VHL and thereby promoted HIF-1α protein stabilization in a pancreatic tumor cell line (24). This mechanism does not appear to operate in macrophages. Hyperactivation of MAPK p38 in *Dusp1*^*−/−*^ mouse BMDMs was accompanied by increased expression of HIF-1α protein (Fig. 1, *C* and *D*) but no increase in the stability of HIF-1α protein (Fig. 1*G*). Likewise, HIF-1α protein stability was not affected by an MAPK p38 inhibitor in human MDMs (Fig. 5*G*).

**Figure 6:**
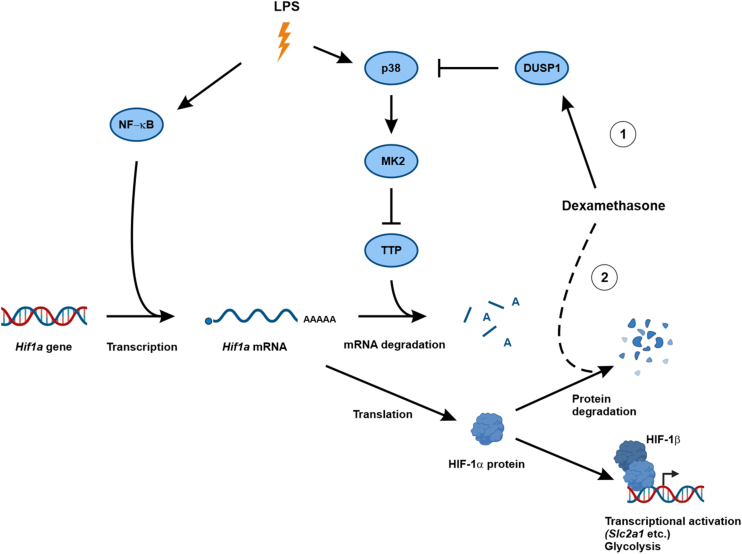
**Regulation of LPS-induced HIF-1α expression by dexamethasone**. LPS promotes accumulation of *Hif1a* mRNA by activating transcription (*e*.*g*., *via* NF- κB) and by inactivating the mRNA destabilizing factor TTP (*via* p38-mediated activation of MK2 and MK2-mediated phosphorylation of TTP Ser52 and Ser178). DUSP1 negatively regulates *Hif1a* mRNA accumulation by preventing the phosphorylation and inactivation of TTP. (1) Although dexamethasone can induce DUSP1 expression, this mechanism is dispensable for the inhibition of LPS-induced HIF-1α accumulation. (2) Dexamethasone promotes destabilization of HIF-1α protein *via* a mechanism that remains to be identified. DUSP1, dual specificity phosphatase 1; HIF-1α, hypoxia-inducible transcription factor 1α; LPS, lipopolysaccharide; TTP, tristetraprolin.

Based on experiments in THP-1 myeloid leukemia cells, Fähling *et al*. (36) concluded that MAPK p38 controls HIF-1α expression *via* the phosphorylation of TTP. It was important to confirm these findings because THP-1 cells are oncogenically transformed and differ from primary macrophages in several respects (50, 51). Using a combination of pharmacological and genetic approaches in primary human and mouse macrophages, here we conclusively demonstrate that MAPK p38 controls HIF-1α expression by modulating the phosphorylation and activity of TTP. Elevated MAPK p38 signaling in *Dusp1*^*−/−*^ BMDMs was accompanied by increased expression of both *Hif1a* mRNA and HIF-1α protein (Fig. 1, *C* and *D*, *E*). This increased expression could be reversed by MAPK p38 inhibition (Fig. 4, *B* and *C*) or by combining *Dusp1* gene disruption with targeted mutagenesis of the *Zfp36* gene, preventing MAPK p38–mediated inactivation of TTP (Fig. 2*E*). In TTP mutant (*Zfp36*^*aa/aa*^) BMDMs, the LPS-induced expression of both *Hif1a* mRNA and HIF-1α protein was impaired (Figs. 2*C*, 4*A*). Furthermore, HIF-1α protein expression was no longer sensitive to MAPK p38 inhibition in these cells (Fig. 4*A*), indicating that MAPK p38 regulates HIF-1α expression principally *via* the phosphorylation of TTP. Collectively, these findings establish the existence of a DUSP1–p38–TTP–HIF-1α regulatory axis (Fig. 6), confirming and extending previous observations (36). This post-transcriptional mechanism of HIF-1α regulation may be relevant in contexts other than macrophages. For example, tumor-suppressive functions of TTP (52) may involve impairment of HIF-1α accumulation, in addition to direct targeting of mRNAs that encode mediators of glycolysis (53, 54). We speculate that, conversely, pro-oncogenic functions of the MAPK p38 pathway (55, 56) involve the inactivation of TTP, promotion of HIF-1α accumulation, and consequent metabolic adaptation to tumor microenvironments.

We considered potential roles of other transcription factors in the regulation of glycolysis by LPS and dexamethasone. The oncogene cMyc promotes glycolysis in many cancers (57) but its function in nontransformed macrophages remains unclear, with reports of both positive and negative roles in glycolysis, depending on the stimulus (58, 59, 60, 61). We found LPS-induced expression of *Myc*/*MYC* mRNA to be extremely transient and dexamethasone insensitive in mouse and human macrophages (our unpublished data), consistent with previous reports of transient induction by LPS of both Myc mRNA and protein (58). We cannot rule out the involvement of cMyc in early responses to LPS treatment. However, cMyc was not required for LPS-induced expression of *Slc2a1* (GLUT1) (58), which was clearly HIF-1α dependent (46). Two additional members of the HIF family were not discussed above. *Epas1*/*EPAS1* mRNA (encoding HIF-2α) was not increased by LPS in mouse or human macrophages, whereas *Hif3a*/*HIF3A* mRNA expression remained exceptionally low under all conditions in mouse and human macrophages (our unpublished data). Together with the powerful inhibition of LPS-induced glycolysis by KC7F2 (26), and of LPS-induced glycolytic gene expression by *Hif1a* knockout (46), these observations support a central role for HIF-1α in LPS-induced glycolysis in macrophages.

The glucocorticoid dexamethasone inhibits HIF-1α accumulation and HIF-1α-directed glycolysis in LPS-treated macrophages (26). We hypothesized that dexamethasone exerts this metabolic effect by activating the well-known glucocorticoid target gene *Dusp1* and thereby inhibiting MAPK p38 function (16, 30, 62, 63). Several pieces of evidence contradicted this hypothesis. In MDMs, an MAPK p38 inhibitor significantly reduced the expression of *Hif1a* mRNA in response to LPS treatment, whereas dexamethasone did not. Dexamethasone destabilized HIF-1α protein in LPS-treated macrophages, whereas an MAPK p38 inhibitor did not. Dexamethasone and the kinase inhibitor cooperated to reduce HIF-1α protein expression in both mouse and human macrophages, supporting the argument that they operate at different levels. Most compellingly, in *Dusp1*^*−/−*^ BMDMs, dexamethasone was still able to inhibit the LPS-induced accumulation of HIF-1α protein, expression of HIF-1α-dependent metabolic genes, and glycolytic reprogramming (Fig. 3). The mechanism(s) by which dexamethasone inhibits LPS-induced HIF-1α expression and glycolytic switch remain(s) to be identified.

The physiological functions of HIF-1α are varied and context dependent, complicating the question of therapeutic targeting of this transcription factor. HIF-1α contributes to the metabolic reprogramming that supports tumor survival and growth in hypoxic environments; therefore, inhibitors are actively sought as anticancer drugs (64). However, the first HIF-targeting drugs to be licensed were PHD inhibitors, which promote the accumulation of HIFs, and are used in the treatment of anemia in chronic kidney disease. HIF-1α has protective, homeostatic functions in the gastrointestinal tract, where its activation by commensal microbes contributes to the maintenance of gut barrier integrity (65). On the other hand, HIF-1α is thought to contribute to the pathogenesis of chronic immune-mediated inflammatory diseases, such as rheumatoid arthritis (66) and cardiovascular disease (67). In the context of host defense, the effects of HIF-1α are two edged. Several reports have described pathogenic roles in severe coronavirus disease 2019 (68, 69, 70). Yet HIF-1α is essential for defenses against intracellular bacteria (6, 71, 72, 73). Even here, this protective role may be dependent on timing: HIF-1α is protective against early infection of macrophages by *Mycobacterium tuberculosis* but drives metabolic changes that support prolonged infection (74).

Where there is strong evidence for a pathogenic role of HIF-1α, our findings suggest that combinations of glucocorticoids with MAPK p38 inhibitors may be effective. Whilst glucocorticoids have powerful anti-inflammatory effects, their prolonged use is associated with many side effects having variable severity (75). In principle, combination with other drugs may allow glucocorticoid sparing and mitigation of such side effects. MAPK p38 clearly contributes to the pathogenesis of many immune-mediated inflammatory diseases, yet the deployment of selective inhibitors as anti-inflammatory drugs has proven difficult. In several clinical trials of MAPK p38 inhibitors, therapeutic anti-inflammatory effects were transient and accompanied by side effects that were likely mechanism related (76). Combination with glucocorticoids could permit therapeutic effects to be achieved with lower doses of inhibitors, minimizing both side effects and tachyphylaxis. These concepts remain to be explored.

## Experimental procedures

### Macrophage isolation and culture

The generation of *Zfp36*^*aa/aa*^ mouse strain was described previously (39). Acquisition of *Dusp1*^*−/−*^ line and the double-targeted *Dusp1*^*−/−*^*Zfp36*^*aa/aa*^ line was also described previously (32). Mice were housed at the University of Birmingham Biomedical Services unit. All maintenance and procedures were carried out according to the Home Office guidelines and approved by the University of Birmingham Animal Welfare and Ethical Review Board.

Mice were sacrificed by cervical dislocation, and their hind legs were removed. For bone marrow isolation, the femur and tibiae were cleaned of muscle tissue, cut at each end, and centrifuged. Extracted bone marrow was placed in RPMI1640 + l-glutamine (Gibco; catalog no.: 21875034) supplemented with 10% heat-inactivated fetal calf serum (Sigma–Aldrich; catalog no.: F2442) and 50 ng/ml macrophage colony-stimulating factor (PeproTech; catalog no.: 300-25) for 7 days to differentiate BMDMs.

Blood from anonymous healthy human donors was obtained as leukapheresis cones from the National Health Service Blood and Transplant Service (ethical approval: ERN_16-0191). Monocytes were isolated using Ficoll density gradient centrifugation and negative selection with the RosetteSep Human Monocyte Enrichment Cocktail (STEMCELL; catalog no.: 15068). Isolated monocytes were placed in RPMI1640 + l-glutamine (Gibco; catalog no.: 21875034) supplemented with 5% heat-inactivated fetal calf serum (LabTech; catalog no.: 80837) and 50 ng/ml macrophage colony-stimulating factor (PeproTech; catalog no.: 300-25) for 7 days to differentiate MDMs. We have previously shown that the aforementioned methods of macrophage generation yielded populations of primary macrophages that were >95% pure (77).

For stimulations, macrophages were seeded at 1 × 10^6^ cells/ml (mouse) or 0.5 × 10^6^ cells/ml (human) in 12-well or 6-well tissue culture–treated plates. Stimulation medium was prepared with final concentrations of 10 ng/ml LPS (*Escherichia coli*, Serotype EK100 [Ra] [TLRgrade]—Enzo Life Sciences; catalog no.: ALX-581-010-L002) with/without 100 nM dexamethasone (Sigma–Aldrich; catalog no.: D18893) or 1 μM VX-745 (Stratech; catalog no.: A8686-APE) and/or 1 μM SB202190 (Stratech; catalog no.: A1632-APE), unless otherwise specified. Dexamethasone, VX-745, and SB202190 were dissolved in dimethyl sulfoxide. Dimethyl sulfoxide concentrations were matched across all conditions. For MAPK p38 inhibition experiments, cells were pretreated with VX-745 or SB202190 or vehicle for 30 min prior to LPS addition to ensure effective kinase inhibition.

### Lactate–glucose measurements

Lactate and glucose concentrations from BMDM cell cultures were measured using the Nova Stat Profile Prime cell culture analyzer. Glucose consumption was calculated by subtracting the glucose concentration of RPMI1640 (11.11 mM) from values obtained from conditioned medium samples.

### Seahorse metabolic flux assays

MDMs were seeded at 50,000 cells/well in Agilent Seahorse XFe96 cell culture microplates and left to adhere overnight. As described (78), we used a combined version of Mito and Glyco stress tests. Seahorse XF RPMI medium, pH 7.4 (Agilent; catalog no.: 103576-100) was supplemented with 2 mM l-glutamine (Sigma–Aldrich; catalog no.: G7513). The following injection protocol was used (final assay concentrations): (A) d-glucose (10 mM, Sigma–Aldrich; catalog no.: G7021), (B) oligomycin A (1 μM, Cayman Chemical Company; catalog no.: 11342), (C) carbonyl cyanide p-trifluoromethoxyphenylhydrazone (5 μM, Cayman Chemical Company; catalog no.: 15218) + sodium pyruvate (1 mM, Sigma–Aldrich; catalog no.: P5280), and (D) rotenone (100 nM, Cayman Chemical Company; catalog no.: 83-79-4) + antimycin A (1 μM, Sigma–Aldrich; catalog no.: A8674) + 2-deoxy d-glucose (20 mM, Sigma–Aldrich; catalog no.: D8375).

For Seahorse normalization, immediately after assay completion, cells were incubated with 1 μM calcein-AM viability dye (eBioscience, catalog no.: 65-0853-78) in PBS for 30 min at 37 °C. Fluorescence was measured using a plate reader (excitation, 490 nm; emission, 515 nm), and the viable cell count ratio was calculated.

### Western blotting

Cells were lysed directly into 2X XT Sample buffer (Bio-Rad; catalog no.: 1610791) and 1X XT Reducing Agent (Bio-Rad; catalog no.: 1610792) and passed through a QIAshredder column to remove genomic DNA (QIAGEN; catalog no.: 79656). Samples were then heated to 95 °C for 5 min, and an equal volume of each sample was loaded onto an XT Bis–Tris protein gel (Bio-Rad). XT MES running buffer (Bio-Rad; catalog no.: 1610789) was used to run the gels for 90 min at 100 V. Protein was transferred onto Bio-Rad Trans-Blot polyvinylidene difluoride membranes (Bio-Rad; catalog no.: 1704157) using the Bio-Rad Trans-Blot Turbo transfer system. Primary antibodies were incubated overnight at 4 °C. Horseradish peroxidase–conjugated secondary antibodies were applied for 1 h at room temperature (Cell Signaling Technologies; catalog nos.: 7074 and 7076). Blots were imaged using Clarity Enhanced Chemiluminescence substrate (Bio-Rad; catalog no.: 1705061) and the ChemiDoc MP Imaging System (Bio-Rad). Blots were quantified *via* densitometry using ImageJ Fiji. The following primary antibodies were used: mouse HIF-1α (Cell Signaling catalog no.: 14179); human HIF-1α (BD Biosciences; catalog no.: 610959); phospho-p38(Thr180/Tyr182) (Cell Signaling; catalog no.: 9211); DUSP1 (Cell Signaling; catalog no.: 35217); TTP (Cell Signaling; catalog no.: 71632); GLUT1 (Abcam; catalog no.: ERP3915); MCT4 (Novus Biologicals; catalog no.: NBP1-81251); and α-tubulin (Sigma–Aldrich; catalog no.: T9026).

### RT–quantitative PCR

RNA was isolated using Norgen Total RNA Purification Plus kits (Geneflow; catalog no.: P4-0016) according to the manufacturer’s instructions. RNA concentrations were quantified using ThermoFisher Nanodrop, and only samples with A260:A280 (>1.8) and A260:A230 (>1.0) ratios were used for complementary DNA synthesis. iScript Reverse Transcriptase (Bio-Rad; catalog no.: 1708891) was used to synthesize complementary DNA from 250 ng or 500 ng RNA/reaction. Gene expression was measured by RT–quantitative PCR using the Bio-Rad CFX384 system.

SYBR TB Green Premix Ex Taq (Takara; catalog no.: RR820W) and primers supplied by Sigma–Aldrich were used. Either *UBC* (human) or *Rpl13a* (mouse) was used to normalize mRNA measurements by the 2^−ΔΔCt^ method. Primer sequences (all listed as 5′-3′ sequence, forward then reverse): *Hif1a* primary transcript TGGATGCCGGTGAGTTCTA, ATGGACACATACACACACCACTC; *Slc2a1* GACGATCTGAGCTACGGGGT, GAACTCCTCAATAACCTTCTGGG; *Slc16a3* TTAAAGTCGCCCCCGGC, ATGGTGTGCTGCCAAACAGT; *Tpi1* GTCAATGATGGGGTGGCTCA, GCAGTGCTCATTGTTTGGCA; *Rpl13a* GCGGATGAATACCAACCCCT, CCACCATCCGCTTTTTCTTGT; *HIF1A* GTTTACTAAAGGACAAGTCAC, TTCTGTTTGTTGAAGGGAG; *SLC2A1* GAACTCTTCAGCCAGGGTCC, ACCACACAGTTGCTCCACAT; *UBC* CGGGATTTGGGTCGCAGTTCTTG, CGATGGTGTCACTGGGCTCAAC.

To measure mature mouse *Hif1α* gene expression. A TaqMan gene expression assay (ThermoFisher, assay ID: Mm00468869_m1) was used and normalized to *Rpl13a* (ThermoFisher, assay ID: Mm05910660_g1). The TaqMan assay utilized Applied Biosystem’s TaqMan Gene Expression Master Mix (catalog no.: 4369016).

### Statistical analyses

Statistical tests were performed using GraphPad Prism (GraphPad Software, Inc). For comparison of multiple conditions, one-way or two-way ANOVA was used with appropriate corrections (Dunnett's test, Tukey's test, or Sidak's test) as indicated in figure legends. For comparison of two conditions, we used an unpaired, two-tailed *t* test. All experiments were repeated a minimum of three times, with the exception of the Western blot in Figure 2*B*. This was done only twice for this article, but five other replicates of an identical experiment were reported in our previous publication (32). All figures display the SD of the indicated number of independent experimental replicates. Statistical tests of quantitative PCR data were performed on ΔCt values and not fold differences.

## Data availability

All relevant data are included within the article.

## Supporting information

This article contains supporting information.

## Conflict of interests

The authors declare that they have no conflicts of interest with the contents of this article.
