# Supplementary material for: Lipopolysaccharide induces HIF-1α accumulation via MAPK p38–mediated mRNA stabilization and dexamethasone-sensitive protein stabilization
Source: J Biol Chem. 2025 Dec 23;302(2):111094. doi: 10.1016/j.jbc.2025.111094 (PMC12856306; doi:10.1016/j.jbc.2025.111094)
Supplement: Supplementary Material 1 [file mmc1.docx]

**Lockwood et al. Supporting Information**


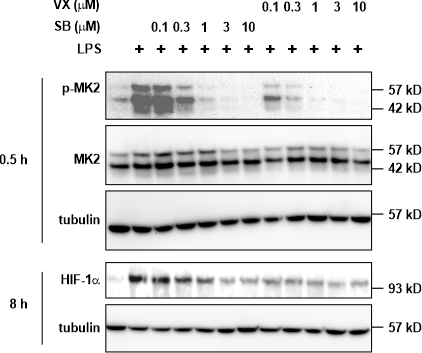


**Lockwood Fig. S1**

Murine BMDMs were pretreated with vehicle (0.1% DMSO) or with the indicated concentrations of SB202190 (SB) or VX-475 (VX) for 0.5 h then stimulated with 10 ng/ml LPS for 0.5 h (upper panels) or 8 h (lower panels). Whole cell lysates were western blotted for total MK2 and phosphorylated MK2 as a readout of MAPK p38 activity (upper) or HIF-1α (lower). In each case tubulin was blotted as a loading control. Representative of three independent experiments.


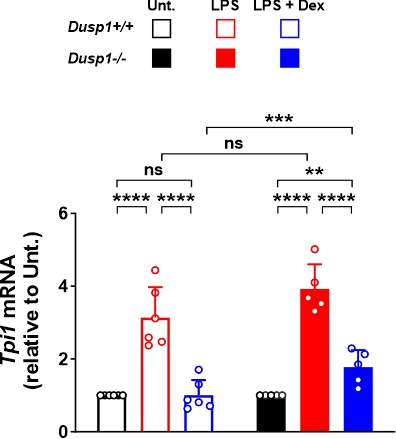


**Lockwood Fig. S2**

As in Fig. 3D, *Dusp1+/+* and *Dusp1-/-* BMDMs were treated for 24 h with 10 ng/ml LPS in the absence or presence of 100 nM dexamethasone (Dex) and *Tpi1* mRNA was measured by qPCR (mean fold change relative to unstimulated control ± SD; five-six independent experiments; two way ANOVA with Tukey’s correction). n.s., p > 0.05; **, p < 0.01; ***, p < 0.005; ****, p < 0.001.
